# Altered Cholesterol Homeostasis in Huntington’s Disease

**DOI:** 10.3389/fnagi.2022.797220

**Published:** 2022-04-19

**Authors:** Radhia Kacher, Coline Mounier, Jocelyne Caboche, Sandrine Betuing

**Affiliations:** ^1^Institut du Cerveau - Paris Brain Institute (ICM), AP-HP, INSERM, CNRS, University Hospital Pitié-Salpêtrière, Sorbonne Université, Paris, France; ^2^INSERM, U1216, Grenoble Institut Neurosciences, Université Grenoble Alpes, Grenoble, France; ^3^Neuroscience Paris Seine, Institut de Biologie Paris-Seine, Faculté des Sciences et Ingénierie, Sorbonne Université, Paris, France; ^4^Centre National de la Recherche Scientifique, UMR 8246, Paris, France; ^5^U1130, Institut National de la Santé et de la Recherche Médicale, Paris, France

**Keywords:** CYP46A1, cholesterol 24-hydroxylase, cholesterol, astrocytes, neurons, therapy

## Abstract

Huntington’s disease (HD) is an autosomal dominant genetic disorder caused by an expansion of the CAG repeat in the first exon of Huntingtin’s gene. The associated neurodegeneration mainly affects the striatum and the cortex at early stages and progressively spreads to other brain structures. Targeting HD at its earlier stages is under intense investigation. Numerous drugs were tested, with a rate of success of only 3.5% approved molecules used as symptomatic treatment. The restoration of cholesterol metabolism, which is central to the brain homeostasis and strongly altered in HD, could be an interesting disease-modifying strategy. Cholesterol is an essential membrane component in the central nervous system (CNS); alterations of its homeostasis have deleterious consequences on neuronal functions. The levels of several sterols, upstream of cholesterol, are markedly decreased within the striatum of HD mouse model. Transcription of cholesterol biosynthetic genes is reduced in HD cell and mouse models as well as post-mortem striatal and cortical tissues from HD patients. Since the dynamic of brain cholesterol metabolism is complex, it is essential to establish the best method to target it in HD. Cholesterol, which does not cross the blood-brain-barrier, is locally synthesized and renewed within the brain. All cell types in the CNS synthesize cholesterol during development but as they progress through adulthood, neurons down-regulate their cholesterol synthesis and turn to astrocytes for their full supply. Cellular levels of cholesterol reflect the dynamic balance between synthesis, uptake and export, all integrated into the context of the cross talk between neurons and glial cells. In this review, we describe the latest advances regarding the role of cholesterol deregulation in neuronal functions and how this could be a determinant factor in neuronal degeneration and HD progression. The pathways and major mechanisms by which cholesterol and sterols are regulated in the CNS will be described. From this overview, we discuss the main clinical strategies for manipulating cholesterol metabolism in the CNS, and how to reinstate a proper balance in HD.

## Introduction

Huntington’s disease (HD) is a hereditary neurodegenerative disease, one of the most frequent genetic brain disorder with a triad of symptoms: movement disorders, cognitive and psychiatric manifestations. Movement disorders, characterized by extrapyramidal signs of chorea, dystonia and bradykinesia, occur early in the course of the disease, and it is preceded by subtle cognitive or behavioral disturbance before the onset of these motor signs (Ross and Tabrizi, 2011). Later, motor impairments include bradykinesia, rigidity and incoordination. HD has a single genetic cause and its transmission is autosomal-dominant with a complete penetrance. The prevalence of HD is about 10 per 100,000 births, with a higher prevalence in some regions of the world (up to 700 per 100,000) (Harper, 1992; Paradisi et al., 2008). The age of onset is usually mid-life, with about 15% of patients showing symptoms before 30 years of age (referred to as juvenile HD). Once the first symptoms have appeared, they progress inexorably and irreversibly over 15–20 years. The availability of informative pre-manifest genetic and neuropsychological testing, along with peripheral and neuroimaging biomarkers of HD progression, offer a window of therapeutic intervention at early stages of the disease, with the perspective to delay the onset of the disease, slow its progression and even prevent HD.

There is no available drug therapy, or gene therapy for slowing disease progression. Over the past two decades, 99 clinical trials were performed in HD investigating 41 different compounds. However, the success rate is low with only 3.5% of trials progressing to the next stage (Travessa et al., 2017). Currently there are 23 active clinical trials in HD registered with ClinicalTrials.gov. Targeting the genetic cause of HD by lowering the product of huntingtin gene (*HTT)* or specifically the harmful *HTT* is still promising but preclinical findings are still essential to open new windows to treat HD by focusing on the cellular consequences of mutant HTT (mHTT) expression. In this regard, several neuronal dysfunctions have been described and could be targeted in clinical assays: excitotoxicity, transcriptional deregulation, dopaminergic alteration, autophagy, loss of neurotrophic support, mitochondrial dysfunction, oxidative stress, and neuroinflammation (Saudou and Humbert, 2016). Studies in humans and mouse models deeply described changes in cholesterol metabolism (Trushina et al., 2006; del Toro et al., 2010; Valenza et al., 2010; Leoni and Caccia, 2015; Boussicault et al., 2016; Kacher et al., 2019) that appear to be a seminal and early event in HD. Hence, targeting cholesterol homeostasis has emerged as an interesting therapeutic approach in HD. In this review, we summarize informative research on cholesterol metabolism in HD before going through the specific role of astrocytes and microglia in cholesterol metabolism deregulation. We address the main clinical strategies to manipulate cholesterol metabolism and we list the pressing questions to optimize the success of a cholesterol-based therapy in HD.

## Huntington’s Disease

The mutation responsible for HD consists of an unstable CAG repeat located at the 5′ end of *HTT* gene on chromosome 4p16.3 (The Huntington’s Disease Collaborative Research Group, 1993). *HTT* gene is non-pathogenic when it contains less than 27 CAG repeats. Between 27 and 35 CAG, repeats do not cause HD but may expand in successive generations. Intermediate allele repetitions (36–39) are associated with late-onset and may express a variable penetrance and different clinical presentation. Individuals with 40 CAG repeats or more will develop HD, with nearly full penetrance by age 65 years (Langbehn et al., 2004). The age of onset inversely correlates with the number of repeats (Bates et al., 2015; Paulson, 2018). However, a large genome-wide association study, showed that genetic factors might influence the onset of HD; specifically these factors could explain the variability of disease onset for a given repeat size (Genetic Modifiers of Huntington’s Disease (GeM-HD) Consortium, 2015, 2019).

The mutation in *HTT* results in an abnormal expansion of polyglutamine (polyQ) in the N-terminal region of the Huntingtin protein (HTT). This mutation causes neuronal dysfunction and death, particularly in the striatum and cortex, despite similar expression of the protein in other brain areas (Ross and Tabrizi, 2011). As neurodegeneration progresses in the striatum, the severity of symptoms increases, and imaging biomarkers of HD progression indicates that striatal atrophy begins up to 15 years before predicted onset (Aylward et al., 2011). Thinning of the cortex, which contains neurons projecting to the striatum, occurs in early symptomatic patients (Rosas et al., 2008), probably as a consequence of neurodevelopmental abnormalities (Barnat et al., 2020). In addition to the gray matter atrophy, several functional magnetic resonance imaging studies showed widespread atrophy of the white matter including from striatal projection fibers and corpus callosum in premanifest HD patients (Dumas et al., 2012; Gregory et al., 2020).

Macroscopic and microscopic criteria have defined a gradation, ranging from grades 0–4, showing a close correlation with the extent of clinical disability and a progressive atrophy of striatal neurons (Vonsattel’s grade) (Vonsattel et al., 1985). The most dramatic degeneration occurs in the medium spiny neurons (MSNs) of the striato-pallidal pathway, which express dopamine receptors of the D2 subtype (indirect pathway; grade 2). This initial loss leads to a hyperkinetic phenotype. Then striato-nigral MSNs, which express dopaminergic D1 receptors (direct pathway), degenerate (grade 3), resulting in bradykinesia. The striatal interneurons, aspiny striatal cholinergic, and somatostatine containing neurons are relatively spared (Roze et al., 2011). Dopamine D2 receptors, which are selectively expressed by indirect but not direct MSNs may be a key factor in the early and selective vulnerability of the indirect pathway and have been implicated in HD pathogenesis (Charvin et al., 2005; Deyts et al., 2009).

Whether neuronal degeneration in HD is due to the loss of one normal *HTT* allele or gain of toxic functions due to *mHTT* on this allele, or both, is not fully elucidated. Knockout of *HTT* in mice is lethal (embryonic day 8.5) (Duyao et al., 1995; Zeitlin et al., 1995), while selective knockout of *HTT* in neuronal cells and testis produces apoptosis in these tissues (Dragatsis et al., 2000), indicating that the HTT protein is required for normal cell development and survival. HTT is a ubiquitous protein, present in all tissues, and within cells, in virtually all cellular compartments. HTT is a large protein of 3,144 amino acids (348-kDa) with numerous intracellular interactors (more than 350), which implicates HTT in diverse cellular functions, including transcription, intracellular transport, metabolism, and homeostasis (Saudou and Humbert, 2016). The interaction with these partner proteins is altered by the mutation, with either enhanced or decreased functions.

Multiple post-translational modifications (PTM) can also affect HTT and/or mHTT functions (Saudou and Humbert, 2016). These include phosphorylation, acetylation, palmitoylation, ubiquitylation, and sumoylation. In the context of mHTT, PTM modulate the toxicity of the protein. For example, acetylation increases clearance by the autophagic-lysosomal pathway and decreases toxicity. Phosphorylation on diverse amino acids within the protein regulates the nuclear transport of mHTT, thus reducing its local toxic functions; this PTM can also decrease the proteolysis of mHTT, or regulates microtubule-dependent transport of organelles. In addition, recent evidence indicate that HTT can regulate the activity of palmitoyl-acyl transferases (PATs), via protein-protein interactions, and increasing brain palmitoylation restores neuropathology, locomotor deficits, and anxio-depressive behaviors in an HD knock-in mouse model (Virlogeux et al., 2021).

Huntingtin is subjected to proteolysis at multiple sites by a variety of proteases, including caspases, calpain, cathepsins, and the metalloproteinase MMP10 (Saudou and Humbert, 2016). The activity of these proteases is increased in the brain of HD patients, resulting in enhanced proteolysis of mHTT. Consequently, small N-terminal fragments containing the polyQ stretch accumulates. These cleaved versions give rise to insoluble aggregates and accumulate in the brain of HD patients and in various HD mouse models (DiFiglia et al., 1997; Scherzinger et al., 1997). Of interest, they first appear in striatal neurons, more abundantly in the neuronal processes and axonal terminals, where they induce altered synaptic functions and neuritic degeneration (Li et al., 2000), thereby eliciting a microglial reaction and inflammatory cytokines production (Yang et al., 2017). In other cellular compartments, mHTT sequester the wild-type HTT or associated proteins involved in normal cellular functions, including transcription, transport, or the ubiquitin-proteasome machinery (Ross and Tabrizi, 2011; Roze et al., 2011). The machineries of unfolded protein clearance, including the ubiquitin-proteasome and autophagy machineries, are altered by the mutation. As such, the aggregates cannot be properly cleared from the cells. It is noteworthy that inhibiting the aggregation of mHTT can alleviate the symptoms in various models of HD. It is noteworthy that inhibiting the aggregation of mHTT can alleviate the symptoms in various models of HD. Although these results support that mHTT aggregates participate to toxicity, other studies indicate that they may initially play a protective role in cells (Saudou et al., 1998; Arrasate et al., 2004). Indeed, smaller oligomeric aggregates could be more toxic than larger ones (Gong et al., 2008), at least in non-neuritic compartments. *mHTT* can also be mis-spliced to generate a short mRNA, which is translated into a highly toxic N-terminal fragment (Bates et al., 2015).

Due to its polyQ expansion, mHTT also alters protein-protein interactions and cellular functions including transcription (Moumné et al., 2013), axonal transport (Vitet et al., 2020), synaptic transmission (Roze et al., 2011), mitochondrial ATP production (Duan et al., 2014), the ubiquitin-proteasome system, and autophagy (Ortega et al., 2010; Li and Li, 2011; Martin et al., 2015). In the last decade, alteration of cholesterol homeostasis was demonstrated in HD (see below for further details; Karasinska and Hayden, 2011; Valenza and Cattaneo, 2011).

Transcriptional dysregulation is a pivotal feature of HD. In HD patients and experimental (cellular, mouse) models, early and progressive changes in transcriptional profiles occur in the prodromal period and affect multiple genes involved in cell survival, plasticity, neurotransmission, metabolism, and homeostasis. These alterations occur principally in the striatum and cerebral cortex, the most affected brain areas in HD, and result from altered nuclear localization and interactions of mHTT (under its soluble or aggregated form) with transcription factors or co-activators/co-repressors (Moumné et al., 2013). Altered cytoplasmic interaction of mHTT with the transcriptional repressor R element-1 silencing transcription factor (REST), results in a nuclear translocation of REST and its inhibitory role on transcription of survival genes, including Brain Derived Neurotrophic Factor (BDNF) (Zuccato and Cattaneo, 2009). Transcriptional dysregulation also occurs in astrocytes, where downregulation of the glutamate transporter GLT1 occurs because of mHTT expression. This in turn leads to a reduction of glial glutamate uptake, resulting in increased extracellular levels of glutamate and excitotoxicity within the striatum (Roze et al., 2011). With regard to cholesterol metabolism, mHTT reduces nuclear translocation of the sterol regulatory element-binding protein 2 (SREBP2), a master transcriptional regulator of genes involved in the cholesterol biosynthesis pathway in astrocytes (Moumné et al., 2013). It also interferes with the DNA binding of nuclear receptors, liver X receptors (LXR), which participate in the cholesterol metabolism and transport (see below). Besides these transcriptional dysregulations, mHTT also affects the epigenetic status, via altered methylation of DNA, histone PTMs, and non-coding RNAs (Moumné et al., 2013).

Alteration of axonal transport is also an important hallmark in HD. Altered interaction of mHTT with motor proteins has a strong impact on vesicular transport and therefore synaptic dysfunction. More specifically, the transport and release of BDNF, from cortical to striatal neurons, is impaired in HD, thus participating in striatal vulnerability (Vitet et al., 2020).

Clinical, biochemical, and neuroimaging studies in HD brain patients provided evidence for deficits in energy metabolism, reduced glucose consumption, and increased lactate concentrations in the basal ganglia and the cortex (Mochel et al., 2007). Furthermore, 3-Nitropropionic Acid (3-NP), an irreversible inhibitor of the respiratory chain complex II, Succinate Dehydrogenase (SDH), induces clinical and neuropathological features that resemble those described in HD, in patients and in rodent models (Brouillet et al., 2005). The mechanisms underlying the deficit energy in HD include impaired oxidative phosphorylation, oxidative stress, impaired mitochondrial handling and trafficking, along with transcription dysregulation (Johnson et al., 2021). Altogether, these data highlight the molecular and pathological consequences of HTT mutation, thereby offering therapeutic opportunities for treatments.

## Cholesterol in Central Nervous System and Aging

**FIGURE 1:**
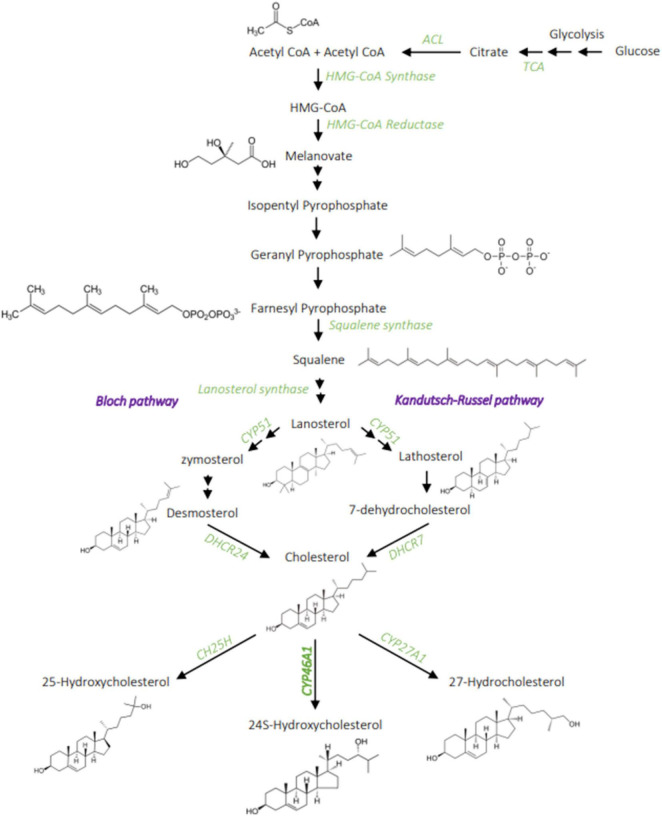
Cholesterol metabolism. Cholesterol synthesis involves a complex chain of enzymatic reaction, from acetyl-CoA to the melanovate pathway that then divides into two possible pathways: the Bloch and Kandutch-Russel. The main product of cholesterol hydroxylation in the brain is the 24S-hydroxycholesterol, it can also be hydroxylated into 27-hydroxycholesterol and 25-hydroxycholesterol to a lesser extent. ACL, ATP citrate lyase; TCA, tricarboxylic acid cycle; CH25H, Cholesterol 25 hydroxylase; CYP51, lanosterol 14 alpha-demethylase; DHCR7, 7-dehydrocholesterol reductase; DHCR24, 24-dehydrocholesterol reductase; CY46A1, cholesterol 24-hydroxylase. Created with BioRender.com.

**FIGURE 2:**
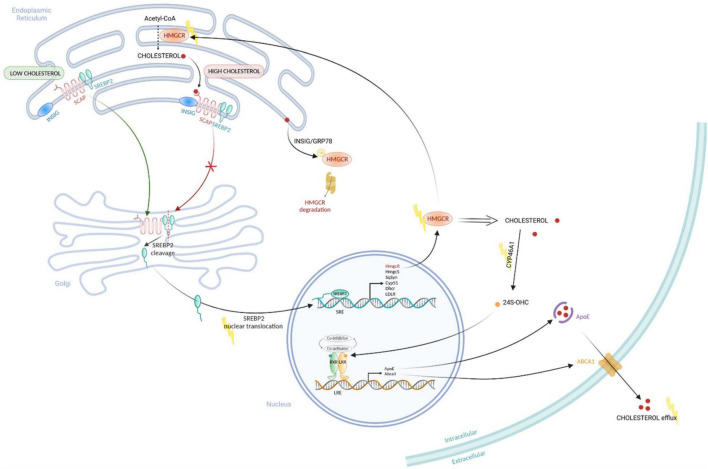
Regulation of Cholesterol metabolism pathways. Cholesterol is synthesized in the endoplasmic reticulum (ER) from Acetyl-CoA through several steps using different enzymes, including the rate limiting enzyme HMGCOA-reductase (HMGCR). SREBP2 forms a complex with SCAP, a sensor of cholesterol, which is anchored at the ER membrane. When cholesterol is high, the sensor SCAP interact to INSIG protein, maintaining the SREBP2/SCAP complex at the ER membrane. When cholesterol level is low, SREBP2/SCAP complex can translocate to the Golgi where SREBP2 is activated by a cleavage. The activated SREBP2 can then translocate to the nucleus to induce the transcription of genes coding cholesterol synthesis enzymes, such as HMGCOA-reductase (HmcR), HMGCO-synthase (HmgcS), Squalen Synthas (SqSyn), Cyp51, Dhcr. This is associated to a negative feedback loop on cholesterol synthesis pathway. Sterol accumulation in the ER membrane triggers proteasome-mediated degradation of HMGCR through INSIG/GRP78-mediated mechanism. In parallel, the enzyme CYP46A1 can catalyze the cholesterol into 24S-OHC, a ligand of the nuclear receptor LXR. When the 24S-OHC binds to LXR, a co-activator is recruited and induce the transcription of genes involved in cholesterol efflux (Abca1, ApoE), leading to a decrease of intracellular cholesterol level. Yellow lightning points out altered functions in cholesterol metabolism in HD. Created with BioRender.com.

In the human brain, cholesterol accounts for 23% of the total body cholesterol, when the brain volume accounts for about 2.1% of the body mass (Dietschy, 2009). Brain cholesterol is mainly unesterified; the larger pool being found in oligodendrocytes myelin sheaths (70% of the brain cholesterol), with a very slow turnover (5 years half-life). The remaining 30% is found in cell membranes, with a faster turnover and a half-life of 5–10 months (Davison, 1965; Andersson et al., 1990). Cholesterol cannot cross the blood-brain barrier (BBB), due to its association with lipoproteins, so it is synthesized locally in the CNS (Jeske and Dietschy, 1980). Newly synthesized cholesterol comes mainly from neurons during embryogenesis then from oligodendrocytes during postnatal myelination, and from astrocytes in the adult brain (Saher and Stumpf, 2015). Cholesterol synthesis in mammals’ CNS involves a complex series of reactions, catalyzed by over 30 enzymes, and requires energy and oxygen (Figure 1; Gaylor, 2002). The first step is the conversion of acetyl-CoA into 3-hydroxyl-3-methylglutaryl-coenzyme A (HMG-CoA) via the reaction catalyzed by HMG-CoA synthetase and then by HMG-CoA reductase (HMGCR) into mevalonate, an irreversible and rate-limiting step in cholesterol synthesis (Rodwell et al., 1976). This is followed by a sequence of enzymatic reactions converting mevalonate into 3-isopenenyl pyrophosphate, farnesyl pyrophosphate, squalene, and lanosterol, followed by 19 steps involving two related pathways. These two pathways for cholesterol production in the brain seemingly have preferential cell expression: neurons preferentially go through the Kandutsch-Russel pathway via synthesis of 7-dehydrocholesterol (7-DHC), and astrocytes preferentially go through the Bloch pathway via synthesis of desmosterol. In the adult brain, the Bloch pathway seems to be preferred (Pfrieger and Ungerer, 2011). Cholesterol is synthesized *via* reduction of 7-DHC by 7-dehydrocholesterol reductase (DHCR7) in the Kandutsch-Russel pathway, and reduction of desmosterol by 24-dehydrocholesterol reductase (DHCR24) in the Bloch pathway (Figure 1). The machinery for cholesterol synthesis resides in the endoplasmic reticulum (ER), where one of its main regulators is SREBP2 (Figure 2), a transcription factor functioning as a sensor of cholesterol in the cell. When inactive, SREBP2 is anchored to the ER membrane and binds to SREBP cleavage activating protein (SCAP). When cholesterol concentration is high in the cell, SCAP, which has a cholesterol-sensing domain, binds to an insulin-induced protein (INSIG), maintaining the SREBP-2/SCAP complex in the ER membrane. When cholesterol levels decrease, SREBP-2/SCAP complex is dissociated from INSIG, allowing SCAP to escort SREBP-2 to the Golgi, where SREBP-2 is cleaved by SCAP. The resulting N-terminus domain of SREBP-2 then translocates into the nucleus and binds to the sterol regulatory elements (SRE) in the promoter region of target genes involved in the synthesis and uptake of cholesterol, fatty acids, triglycerides, and phospholipids (Horton, 2002). Cholesterol biosynthesis has an important feedback control on HMGCR activity and degradation (Goldstein and Brown, 1990). Accumulation of sterols in the ER membranes triggers the proteasome-mediated degradation of HMGCR through an INSIG/GRP78 dependent ubiquitination (Tsai et al., 2012). Thus, cholesterol synthesis is regulated by a negative feedback loop dependent on sterol levels.

The cholesterol brain turn-over is ensured by the cholesterol 24-hydroxylase or CYP46A1, a neuronal-enriched enzyme that converts, in a reaction requiring NADPH, cholesterol into 24S-hydroxycholesterol (24S-OHC), by addition of a hydroxyl group to the lateral hydrocarbon chain of cholesterol (Dhar et al., 1973; Dzeletovic et al., 1995). This conversion allows the crossing of 24S-OHC through the BBB, thus the elimination of cholesterol from the brain. *In vivo* formation of 24S-OHC was measured in the blood in humans (Lütjohann et al., 1996), rats (Breuer and Björkhem, 1995), and mice (Meaney et al., 2000). CYP46A1 is responsible for 99% of 24S-OHC in the brain, which account for 60–80% of 24S-OHC in the serum (Lund et al., 2003). According to the continuous flux of 24S-OHC in the serum (Lütjohann et al., 1996), cholesterol turnover by CYP46A1 in the brain appears to be a daily mechanism, which could account for a daily turnover of 20% in certain neurons (Dietschy and Turley, 2004). Within the brain, 24S-OHC is also a key modulator of the nuclear receptors: Liver X Receptor (LXR), which regulate the transcription of cholesterol transport proteins (see below). To a lesser extent, CYP46A1 can also catalyze the formation of 25-hydroxycholesterol, 24,25-dihydroxycholesterol and 24,27-dihydroxycholesterol (Lund et al., 1999; Mast et al., 2003).

In the CNS, cholesterol has many essential functions in the cells, and particularly in neurons. Due to its rigid apolar ring structure, cholesterol is deeply immersed in membranes reducing membrane dynamic and fluidity (Sankaram and Thompson, 1990a,b). The increase of cholesterol in membranes slows down the lateral diffusion of lipids and proteins (Rubenstein et al., 1979) and therefore can influence the conformation of membrane proteins, changing for instance the stability and binding properties of neurotransmitter receptors with their ligands (Gimpl et al., 1997; Saxena and Chattopadhyay, 2012). Cholesterol has also an essential role in vesicle dynamics and constitutes 40% of total lipids in synaptic vesicle membranes (Takamori et al., 2006). It can modulate the vesicular transport likely through the recruitment of motor proteins (van der Kant et al., 2013), and influence fusion pore formation and stability during exocytosis (Koseoglu et al., 2011), by directly reducing the fusion pore-bending energy (Stratton et al., 2016). In this way, cholesterol depletion by β-cyclodextrin increases the spontaneous neurotransmission in hippocampal cultures, due to an enhanced spontaneous vesicle recycling (Wasser et al., 2007). In a recent study, cholesterol was shown to be an important endogenous regulator of synaptic transmission by acting on the post-synapse (Korinek et al., 2020). Within the bilayer membrane, cholesterol is enriched in lipid rafts, which participate in the compartmentation of proteins involved in the spatial and temporal organization of cell signaling, and as such have a critical role in the signal integration at the synapse; they are likely associated with the organization of post-synaptic density (Suzuki et al., 2011). Acute cholesterol depletion decreases both *N*-methyl-D-aspartate receptor (NMDA) and α-amino-3-hydroxy-5-methyl-4-isoxazolepropionic acid receptors (AMPA)/kainate receptor-mediated evoked excitatory postsynaptic currents and impairs NMDA-dependent long-term potentiation (LTP) (Korinek et al., 2020). Moreover, alteration of lipid rafts by cholesterol depletion suppresses AMPA receptor exocytosis, reducing its cell surface expression (Hou et al., 2008), and produces a gradual loss of synapses and dendritic spines (Hering et al., 2003).

Besides its role at post-synaptic membranes, cholesterol accumulation at lysosomal membranes reduces the lysosome ability to fuse with endocytic and autophagic vesicles, thus affecting autophagy, *via* a process involving abnormal sequestration of SNARE proteins in cholesterol-enriched regions of endolysosomal membranes (Fraldi et al., 2010). Cholesterol distribution and regionalization within synaptic vesicles modulate SNARE protein conformations and complex assembly (Wang et al., 2020). An essential role of cholesterol in vesicle trafficking comes from studies on Niemann-Pick disease (NPC), a lysosomal storage disease characterized by lysosomal accumulation of unesterified cholesterol and glycosphingolipids, which results in alteration of axonal lysosome trafficking and positioning in neurons (Roney et al., 2021). In a cellular model of NPC, lowering lysosomal cholesterol levels with cyclodextrin reduces lysosome transport into NPC axons and reduces axonal autophagic stress. Therefore, cholesterol is an essential structural component for the integration of intracellular signaling through the organization of the plasma membrane in micro-domains like lipid rafts, it regulates the dynamic of synaptic vesicle biogenesis and vesicle fusion, thus participating to the proper transport of vesicles along microtubules.

Because of its critical role in membrane dynamics and cell signaling, alterations of cholesterol content and homeostasis can have deleterious impacts on cell survival. The binding of BDNF to its cognate TrkB receptor triggers activation of two major survival intracellular signaling pathways: the Extracellular-signal Regulated Kinase (ERK) and Akt pathways. Stimulation of cultured neurons and hippocampal slices by BDNF induces an increase of TrkB receptor location in lipid rafts, showing a potential need for selective receptor location for efficient signaling (Suzuki et al., 2011). Cholesterol overload can decrease TrkB signaling and cell survival (Huang et al., 2016), and, in aging neurons, loss of cholesterol is associated with increased TrkB activity (Martin et al., 2008) while knockdown of CYP46A1 in neurons triggers low TrkB activity and increased apoptotic levels (Martin et al., 2011). Identification of cholesterol-interacting sequences (CRAC and CARC) was reported in TrkB sequence and their mutation can interfere with plasticity-related BDNF signaling (Cannarozzo et al., 2021; Casarotto et al., 2021). Because of their high need for energy production, neurons progressively accumulate reactive species of oxygen (ROS). In conditions of oxidative stress-related production of ROS, loss of membrane cholesterol is associated to the activation of TrkA, which binds Nerve Growth Factor (NGF) another prosurvival Growth Factor. Addition of cholesterol in non-stressed condition inhibits activation of TrkA and favors stress (Iannilli et al., 2011). Thus, changes in cholesterol content might be a way to regulate receptor activity toward cell surviving pathways, acting as a sensor of the cell state. Altogether, cholesterol level seems to be precisely regulated to favor cell survival, within a physiological range of concentration.

To understand pathological alterations of cholesterol metabolism, it is necessary to understand its evolution during normal aging. Alteration of cholesterol content is highly variable during aging in the different brain regions, ranging from negligible changes in the hippocampus and pons to a 40% decrease in the caudate at 90 years old (Söderberg et al., 1990). On average, cholesterol decreases by 47% in women and 53% in men, with a significant loss of myelin lipids (Svennerholm et al., 1997). Cholesterol loss is more pronounced within the white matter than in the cerebral cortex (Svennerholm et al., 1991). Cholesterol synthesis rate declines with aging in the human hippocampus, with a lower concentration of lanosterol and desmosterol precursors in elderly subjects (Thelen et al., 2006). A decreased synthesis with altered expression of genes related to cholesterol synthesis also occurs in the aging rat brain (Blalock et al., 2003). In the aging rat hippocampus, lanosterol, desmosterol, and lathosterol levels decrease, whereas in the cortex, only desmosterol levels decrease over time (Smiljanic et al., 2013). The mouse hippocampus shows an age-dependent loss of cholesterol (Martin et al., 2008; Sodero et al., 2011) which is associated with a cognitive decline (Martin et al., 2014). Plasma levels of 24S-OHC are very high in children, then decreases to become constant during adult life and tends to increase with age (Bretillon et al., 2000). The exact causes of this loss are not yet clear, but some mechanisms were proposed. Among them, an increase of transcriptional activation (Ohyama et al., 2006) and membrane mobilization (Sodero et al., 2012) of CYP46A1 could participate in cholesterol loss during aging. Particularly, CYP46A1 increases in high-stress situations, accumulation of cellular stress over time could favor cholesterol loss in aging. The age-dependent lowering of cholesterol may also be due to reduced synthesis or impaired delivery from glial cells.

## Impairment of Cholesterol in Huntington’s Disease

### Deregulation of Cholesterol Metabolism in Huntington’s Disease

An increasing number of studies have implicated dysregulations of cholesterol metabolism in HD (see Table 1 for references). Studies on rodent models, cell lines, and data from patient samples have shown a global downregulation of cholesterol metabolism. The amount of lathosterol and lanosterol decreases in multiple rodent models and patient plasma. This global alteration of the cholesterol biosynthesis pathway could be due to a decreased translocation of the transcription factor SREBP2 to the nucleus and a decreased expression and activity of synthesis enzymes. In the YAC128 HD mouse model, while SREBP2 processing (i.e., cleavage) is not affected, the mature cleaved SREBP2 accumulates in the cytoplasm, due to increased binding of mHTT to the SREBP2/importin β complex required for its nuclear import (Di Pardo et al., 2020). This could explain the reduction of the expression levels of HMGCR, CYP51, DHCR7, and DHCR24 found in HD whole brain extracts and in astrocyte primary cultures (Table 1).

**TABLE 1:** Impairment of cholesterol metabolism in HD models and patients.

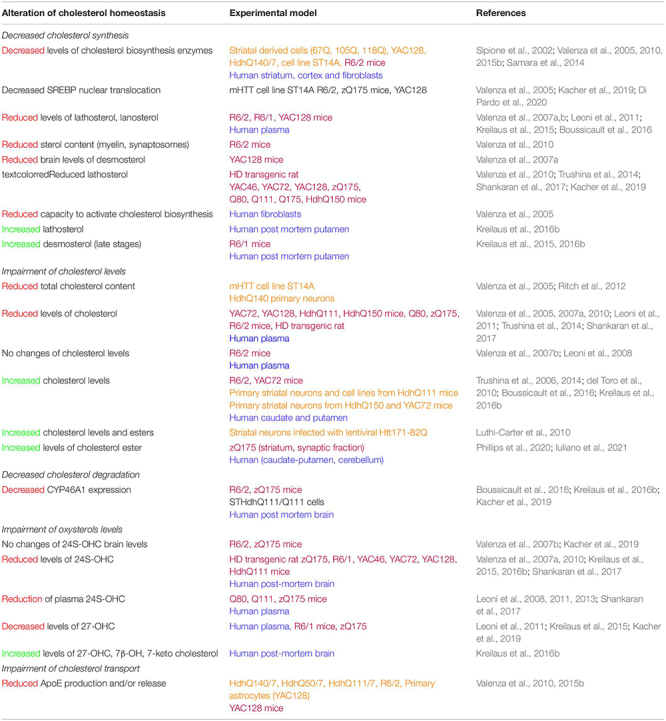

*Rodent models in purple, cell cultures in orange and human samples in blue. Decreased levels are in red, increased levels in green.*

Studies of cholesterol content gave rise to differential results, with decreased, increased, or no changes in cholesterol levels in the brain of HD animal models; whereas studies in human post-mortem caudate and putamen showed increased cholesterol levels (Table 1). Studies on cell cultures showed an accumulation of cholesterol in membranes due to the altered trafficking of cholesterol by caveolin-1 (Table 1 for reference). The methodology used to measure cholesterol levels is critical to have reproducible data. A comparative study demonstrated that biochemical and mass spectrometry methods showed reduced cholesterol levels whereas colorimetric and enzymatic methods showed increased cholesterol levels. Colorimetric and enzymatic methods appear to have a lower sensitivity, giving more variable results than analytical methods like gas chromatography-mass spectrometry. The method for sample preparation is also critical for sensitive and reliable measures (Marullo et al., 2012).

The level of inactive esterified cholesterol, cholesterol esters, is increased in HD mouse models and post-mortem brain tissues (Table 1). This may be a compensatory mechanism to reduce cholesterol accumulation since the conversion in this inactive form facilitates cholesterol transport by increasing the amount of cholesterol packaged in lipoproteins. The product of cholesterol degradation in the brain, 24S-OHC is decreased in the brain of several HD models and patient tissues. Interestingly, 24S-OHC levels decrease in patient plasma at early stages and correlate with motor impairment and caudate atrophy (measured by MRI) (Table 1). 24S-OHC seems to be an interesting biomarker for HD progression, although other peripheral factors have to be considered to measure its accurate levels in the blood, such as plasma lipoproteins turnover and the rate of excretion of oxysterols by the liver (Leoni and Caccia, 2013).

Cholesterol transport by astrocytes is less efficient in HD, with decreased expression of apolipoprotein E (ApoE) and ABCA1. Astrocytes expressing mHTT produce and release less ApoE, impacting cholesterol transport to neurons (Table 1). Reduced transport might be associated with a decreased activity of LXR in the context of HD. Indeed, LXR are nuclear receptors, which bind oxysterols (including 24S-OHC) and desmosterol, and regulate the transcription and expression of protein involved in cholesterol transport like ApoE and ABCA1 (Abildayeva et al., 2006). LXR activation is also important for the expression of the cholesterol synthesis enzymes FDFT1 (Farnesyl-Diphosphate Farnesyltransferase 1) and CYP51 (lanosterol C14 demethylase) (Wang et al., 2008), as well as for oligodendrocyte differentiation and myelination (Xu et al., 2014; Meffre et al., 2015), and modulation of inflammation (Zelcer et al., 2007; Morales et al., 2008). In normal conditions, HTT acts as a co-activator for LXR transcription factors, and HTT mutation can potentially lead to a loss of this function and thus a decreased LXR activity (Futter et al., 2009). The Peroxisome proliferator-activated receptor gamma coactivator-1 alpha (PGC-1 α) is a key protein that regulates energy metabolism and in particular mitochondrial biogenesis. Of interest in HD, mHTT decreased PGC1α expression and knockout of PGC1α causes a reduction of cholesterol synthesis and degradation and is associated with a defect of myelination (Xiang et al., 2011). Especially, a knockout of PGC1α in oligodendrocytes affects cholesterol biosynthetic pathway, by decreasing expression of HMGCS1 and HMGCR (Xiang et al., 2011).

The whole body cholesterol metabolism seems to be altered in HD. Cholesterol metabolism changes may also results of dysfunction related to food digestion and absorption. In this regard, increased intestinal permeability and dysbiosis in the R6/2 mouse model of HD was observed (Kong et al., 2020; Stan et al., 2020). Gut dysbiosis was also shown in HD patients with associations among gut bacteria, cognitive performance and clinical outcomes (Wasser et al., 2020). As the gut-brain axis seems to be important for brain function (Dinan and Cryan, 2017), the involvement of gut dysbiosis in brain cholesterol metabolism dysfunction in HD cannot be excluded.

### Cellular Consequences of Altered Cholesterol Metabolism in Huntington’s Disease

Altered cholesterol metabolism can have deleterious impacts at several levels. Modification of cholesterol content and dynamics can influence membrane fluidity and the distribution of micro-domains. The study of peripheral cells from HD patients showed an alteration of membrane properties and fluidity related to differences in cholesterol and phospholipid content (Muratore, 2013). The number of ordered domains was higher in mHTT expressing neurons, suggesting that cholesterol accumulation is associated with an increased amount of lipid rafts. This was accompanied by an increased localization of NMDA-R in the cholesterol-enriched domains, thus altering NMDA-R distribution, and potentially contributing to NMDA-mediated excitotoxicity. Moreover, administration of cholesterol lowering drugs such as simvastatin and β-cyclodextrin protected against NMDA mediated excitotoxicity (del Toro et al., 2010). Cholesterol can also influence HTT binding and aggregation to membranes, with decreased HTT insertion as cholesterol content increases (Gao et al., 2016).

More recently, analysis of mouse model striatum and post-mortem putamen showed a disrupted localization of synaptic proteins and lipids important for synaptic function, dependent of age, with an altered integrity of synaptic compartments in HD mice (Iuliano et al., 2021). Aberrant interaction between mHTT and caveolin-1 impairs the intracellular trafficking of cholesterol, leading to cholesterol accumulation in the Golgi and lysosomes. This accumulation could affect the normal function of the Golgi apparatus and lysosomes, contributing to cellular toxicity. Interestingly, loss of caveolin-1 in HdhQ150 mouse model was able to rescue cholesterol trafficking and motor decline in these mice (Trushina et al., 2006, 2014).

A proper supply of cholesterol is critical for neurite outgrowth, synapses and dendrites formation, along with axonal guidance (Goritz et al., 2005; Fester et al., 2009). A global alteration of cholesterol metabolism can affect neurotransmission, and cholesterol depletion leads to a decreased synaptic plasticity and neurite degeneration caused by a neurodegenerative break of neurofilaments integrity (Koudinov and Koudinova, 2005). Moreover, targeting cholesterol synthesis by inhibition of HMGCR causes neurite loss by interfering with the melanovate pathway (Schulz et al., 2004). At late stages in the transgenic R6/2 HD mouse model, cholesterol content decreases in myelin, highlighting a potential link between cholesterol impairment and myelin defects in HD (Valenza et al., 2010). Since cholesterol in myelin is critical for efficient transmission of the action potential, this defect could impair neuronal transmission. Thus, a global alteration of cholesterol metabolism can affect neurotransmission.

Studies showed that mHTT decreased mitochondrial membrane fluidity in *STHdh* cells, in isolated mitochondria from HD knock-in mice and BACHD rats, while mitochondrial cholesterol levels only decreased in BACHD rats (Eckmann et al., 2014). The authors concluded that cholesterol levels might not be the only determinant of membrane fluidity changes found in mitochondria isolated from different HD models.

Several strategies can be used to manipulate and modulate the cholesterol metabolism pathway and cholesterol content in order to improve cell survival in HD. For example, the addition of cholesterol rescued the cell death induced by mHTT in human HD cell lines (Valenza et al., 2005) and, when supplemented in nanoparticules *in vivo*, partially alleviated HD phenotype in a mouse model (Valenza et al., 2015a). In HD primary cultures, where mHTT increased cholesterol level, inhibition of sirtuin 2 decreased nuclear trafficking of SREBP-2, cholesterol synthesis and protected from mHTT toxicity (Luthi-Carter et al., 2010). Of interest, addition of cholesterol precursors lanosterol and desmosterol are neuroprotective *in vitro* on primary cultures of striatal neurons expressing mHTT (Boussicault et al., 2016; Kacher et al., 2019), showing a global need to compensate for impaired cholesterol metabolism through cholesterol precursors.

## Role of Astrocytes and Microglia in Huntington’s Disease and Consequences in Cholesterol Dysregulation

### Cellular Dysfunctions in Huntington’s Disease Astrocytes

Astrocytes are critical component of the CNS, where they play an important role in the maintenance of brain homeostasis and neuronal function. They are involved in the neurovascular coupling to ensure the supply of neurons, in maintaining extracellular ion balance, supporting synaptogenesis, supplying nutrients to neurons and have an important role in neurotransmission. Astrocytes regulate the levels of neurotransmitters (Glutamate, GABA) and ion homeostasis at the synapse, they also release gliotransmitters to influence synapse functions (Wilton and Stevens, 2020). Therefore, the alteration of their normal function can affect the global CNS homeostasis and functions. Although neurodegeneration in HD is classically associated with cellular dysfunctions in striatal MSN, the contribution of glial cells in this neuronal pathogenicity must be taken into consideration. Studying glial alteration could also help to understand the region-specific susceptibility observed in HD.

In HD, mHTT aggregates accumulate less in astrocytes than in neurons (Jansen et al., 2017), possibly because of a faster degradation of mHTT in astrocytes (Zhao et al., 2016). However, several cellular alterations are found in HD astrocytes, such as a reduction of astrocyte surface area, alteration of differentiation, reduced association with neuronal connexions, altered regulation of neurotransmitter release and uptake from synapse, and reduction of intracellular calcium signaling pathway (Wilton and Stevens, 2020). mHTT-induced transcriptional dysregulations can also occur within astrocytes, including a downregulation of mRNA levels coding the glutamate uptake transporter GLT1 and the potassium channel Kir4.1. GLT1 mRNA and protein levels are decreased early in the putamen of HD patients and correlates with disease severity (Arzberger et al., 1997; Behrens et al., 2002). The decrease of astrocytic GLT1 levels in R6/2 mice induces a decrease of glutamate re-uptake, associated with an increased level of extracellular glutamate, which contribute to the excitotoxicity described in striatal neurons. When mHTT is only expressed in astrocytes, GLT1 expression is affected, glutamate uptake is altered, dysfunction of striatal neurons and motor abnormalities are observed (Bradford et al., 2009; Faideau et al., 2010), arguing for an astrocyte specific effect of mHTT in the HD pathogenesis. Interestingly, treating symptomatic R6/2 mice with ceftriaxone, an antibiotic known to increase GLT1 expression, prevents the decrease of glutamate uptake as well as the motor performance deficits (Miller et al., 2008). However, the GLT1 ablation does not worsen motor deficits and weight loss in R6/2 mice (Petr et al., 2013). Protein levels of the glutamate transporter GLAST are also decreased in the striatum and cortex of R6/2 mice (Estrada-Sánchez et al., 2009), associated with neuronal vulnerability, and the selective inhibition of both GLT1 and GLAST in R6/2 brain slice worsen electrophysiological properties of HD cortical neurons (Estrada-Sánchez et al., 2019). The astrocytic Kir4.1 channel has a role in the influx of potassium and therefore it is essential to support MSNs electrophysiological properties (Nwaobi et al., 2016). The reduced expression of Kir4.1 in astrocytes in R6/2 and zQ175 mice induces an increase of striatal extracellular K^+^ level, which might underlie the hyperexcitability of MSNs in HD. The restoration of Kir4.1 channel expression in R6/2 mice, restores normal extracellular K^+^ level, rescues MSN excitability and improves motor phenotype (Tong et al., 2014).

Another important feature in HD is astrocyte reactivity. In response to a homeostatic dysregulation, astrocytes become reactive, and present transcriptional, morphological and functional changes. Reactive astrocytes become hypertrophic and present an increased GFAP (Glial Fibrillary Acidic Protein) expression, which is observed in the striatum of HD mice and in post-mortem striatal tissues from HD patients at early stages. The number of GFAP positive cells correlates with the disease severity, and spread latter in the cortex of HD patients (Faideau et al., 2010). A deleterious role of reactive astrocytes has been shown in several neurodegenerative diseases (Ben Haim et al., 2015a), such as Alzheimer’s disease (AD), where blocking astrocyte reactivity in mice reduces amyloid deposition, improves synaptic functions and spatial learning (Ceyzériat et al., 2018). By contrast, the prevention of astrocyte reactivity in HD, using an endogenous inhibitor of the JAK/STAT3 pathway, increases the number of mHTT aggregates, but does not affect neuronal death (Ben Haim et al., 2015b). In HD mouse model, mHTT nuclear inclusion are less numerous in GFAP-positive astrocytes (4–10%), as compared with S100β astrocytes (30%) (Jansen et al., 2017), which could be explained by an increased proteasome activity in GFAP-positive astrocytes (Orre et al., 2013). These data suggest a protective role of reactive astrocytes in HD, due to their higher ability to clear mHTT aggregates.

Besides astrocyte reactivity, mHTT expression in astrocytes plays a key role in HD pathogenesis, as demonstrated in several studies, which showed that astrocyte-specific expression of mHTT is sufficient to induce age-dependant HD phenotype, including motor dysfunction, weight loss, shortening of life span (Bradford et al., 2009), along with neuronal excitotoxicity in neurons-astrocytes co-cultures (Shin et al., 2005). mHTT expression in astrocytes also induces a decrease of BDNF transcription and release *in vitro* (Wang et al., 2012) due to an impairment of BDNF-containing vesicles exocytosis (Hong et al., 2016). The striatal engraftment of mHTT-expressing astrocytes in WT mice induces some HD phenotype, with an alteration of motor performance and a MSNs hyper-excitability, compared to control (Benraiss et al., 2016). Conversely, the prominent neuronal alterations exhibited by HD models can be prevented or reversed by WT astrocytes, such as mHTT-mediated neurotoxicity *in vitro* (Shin et al., 2005) and the striatal engraftment of WT astrocytes in R6/2 mice slows disease progression, improves electrophysiological and behavioral alterations, restores K^+^ homeostasis and increases cell survival (Benraiss et al., 2016). Altogether, these results show how astrocyte alterations are deleterious for neuronal functions. Recent studies highlighted the importance of astrocyte regional heterogeneity, which might explain the regional specific vulnerability of striatal MSNs. For example, *in vitro* HD striatal astrocytes release higher amount of the pro-inflammatory mediator TNFα, toxic for neurons, compared to HD cortical astrocytes. BDNF treatment is protective for HD cortical and striatal astrocytes, inducing an increase of GLT1 expression, but conditioned medium from HD striatal astrocytes treated with BDNF is protective for HD striatal neurons only (Saba et al., 2020). HD is also associated with several metabolism alterations, including low glucose levels in HD mouse brain (Grafton et al., 1992). Astrocytes take up glucose from the blood, metabolize it into lactate and redistribute it to neurons, to ensure neuronal activity (Pellerin and Magistretti, 1994). Glucose uptake is decreased in HD mouse striatum and *in vitro* overexpression of mHTT in astrocytes indirectly impairs glucose uptake in neurons and triggers oxidative stress (Boussicault et al., 2014). To counteract the low glucose levels in a HD brain, astrocytes adapt by metabolically reprogramming their mitochondria to use endogenous and non-glycolytic metabolites as a substitute for energy production, depending on metabolic pools available in each CNS regions. Indeed, striatal astrocyte mitochondria switch to fatty acid oxidation for fuel, with the cost of inducing the production of ROS, toxic for neurons, whereas astrocyte mitochondria from cerebellum use amino-acids precursors for glucose, not associated with ROS production (Polyzos et al., 2019). This metabolic reprogramming depending on brain region might be another cue to better understand specific region vulnerability of striatal neurons in HD. According to these studies, astrocyte dysfunctions need to be carefully considered as a contributor of HD pathogenesis.

### Cholesterol Metabolism and Astrocytes

As mentioned above, another metabolic pathway ensured by astrocytes is cholesterol metabolism, which is strongly impaired in HD. Cultures of HD astrocytes, from different mouse models, show a downregulation of mRNA levels for cholesterol biosynthesis (HMGCR, CYP51, 7DHCR) and efflux (APOE, ABCA1) genes, associated with a decrease of APOE protein levels and secretion of APOE lipoprotein in the medium. HD astrocyte conditioned medium is detrimental for neurons, and does not support synaptic activity (Valenza et al., 2010, 2015b). Enhancing glial cholesterol biosynthesis or transport, with overexpression in astrocytes of SREBP2 or ABCA1, reverses neuronal dysfunctions in HD (Valenza et al., 2015b), supporting the involvement of astrocytic cholesterol metabolism in neuronal survival. Transcriptional dysregulation occurs in HD astrocytes, and a recent study (Benraiss et al., 2021) investigated the specific cell-type changes in gene expression associated to mHTT in R6/2 and zQ175 mice using fluorescence-activated cell sorting (FACS) followed by RNAseq analysis. Astrocytes from R6/2 mice -which overexpress the cleaved version of mHTT corresponding to the exon 1 of the gene- displayed a downregulation of cholesterol biosynthesis genes (SREBF2, INSIG1, CYP51A1, HMGCR, IDI1, HMGCS2, DHCR24, MVD), cholesterol sensors (INSIG1, INSIG2), and cholesterol uptake (LDLR), while cholesterol efflux (ABCA1) was increased, suggesting an abnormal response of HD astrocytes to cholesterol levels. Noteworthy, the cholesterol pathway genes were not altered in isolated astrocytes from zQ175 mice – which express the full-length mHTT. Comparison of these results to *in vitro* human striatal astrocytes expressing either full-length mHTT or exon1-mHTT showed a similar pattern of genes deregulation, with cholesterol pathway deregulation only in exon1-mHTT human astrocytes. Although these data support the implication of astrocytic cholesterol metabolism dysregulation in HD.

Other cross talks between neurons and astrocytes are necessary for the maintenance of cholesterol homeostasis, and for regulating cholesterol synthesis in neurons. APOE particles from astrocytes to neurons contain, besides cholesterol, non-coding microRNAs that will silence neuronal genes encoding cholesterol biosynthesis enzymes (HMGCR, HMGCS1, CYP51) resulting in a downregulation of *de novo* cholesterol synthesis in neurons (Li et al., 2021). Exploring a link between cholesterol metabolism in astrocytes and BDNF in HD could also help to better understand astrocyte alterations and their involvement in neuronal pathogenesis. Indeed, BDNF stimulates cholesterol biosynthesis and efflux, through ABCA1 and APOE expression in WT astrocytes, and BDNF levels are reduced in HD striatum, due to a decrease of supply from cortical neurons and astrocytes. Altogether, these studies underlie the importance of maintaining a correct cholesterol homeostasis in astrocytes and an efficient neuron-astrocyte cross-talk.

### Cellular Dysfunctions in Huntington’s Disease Microglia

Microglia are the resident immune cells in the brain, they monitor the environment and become activated in response to a stimulus to maintain brain homeostasis. In the resting state, they also play an important role in brain development, neurogenesis, synapse maturation and plasticity, they are able to contact neurons with highly motile processes. When microglia become activated, they ensure a phagocytic role, and are involved in synaptic plasticity, neuronal growth and survival (Wilton and Stevens, 2020). Activated microglia have been largely described for their inflammatory function; they can either be neurotoxic or protective depending on the factors released, the duration of activation and the state of activation: pro-inflammatory M1 microglia or pro-repair anti-inflammatory M2 microglia (Yang et al., 2017).

Huntington’s disease is associated with several microglial dysfunctions. mHTT inclusions are present in a very few proportion of microglia from the striatum and the frontal cortex in R6/2 and zQ175 mice (Jansen et al., 2017). However, microglia activation is observed in HD patient post-mortem brains (Singhrao et al., 1999), increasing with disease severity, and correlates with neuronal loss suggesting an involvement of microglia in HD pathogenesis (Sapp et al., 2001). A significant increase of microglial activation was observed by Positron Emission Tomography (PET) in the striatum of HD patients, correlating with HD severity and striatal D2 MSNs dysfunction (Pavese et al., 2006). However, this signature in brain patients seems to be dependent on the PET methodology used. Indeed, microglia activation was detected by PET in pre-symptomatic gene carriers patients, prior to symptom onset (Tai et al., 2007; Politis et al., 2010, 2015), suggesting that it is an early event in HD. Conversely, in a recent PET study using another type of radio-ligand (Rocha et al., 2021), microglia activation in basal ganglia of HD patients occurred later in the course of the disease. HD microglia display morphology changes and reduced motility, which is essential for their monitoring functions (Wilton and Stevens, 2020). Differences in the disease stages of HD microglia alteration are also described between various HD mouse models. FACS isolated microglia from R6/2 mice display transcriptional alterations early in the disease that increase with age, while transcriptional perturbations in zQ175 mice appear latter. These transcriptional modifications are associated to different cellular pathways, with an alteration of inflammation genes pathway in R6/2 microglia, and an alteration of genes associated to cell–cell contact and morphology in zQ175 microglia (Benraiss et al., 2021).

Huntington’s disease microglia is highly associated with a pro-inflammatory phenotype, deleterious for neurons. The levels of pro-inflammatory cytokines IL-6, IL-8, TNFα increase with disease progression in the plasma and cerebrospinal fluid of pre-manifest carrier gene patients (mean of 16 years before symptom onset) (Björkqvist et al., 2008), and are considered as an early event in HD. In the plasma of HD patients, the levels of two anti-inflammatory cytokine (IL-4, IL-10) increase significantly later in the disease, i.e., in moderate stages, suggesting an adaptive response to the early pro-inflammatory response (Björkqvist et al., 2008). Pro-inflammatory mediator levels follow a brain specificity, with an up-regulation of IL-6, IL-8, TNFα, CCL2, IL-10, MMP9 in port-mortem HD human striatal tissue, but only IL-6, IL-8 and MMP9 in the cortex and cerebellum, CCL2 (Björkqvist et al., 2008; Silvestroni et al., 2009). The upregulation of pro-inflammatory cytokines has been also reported in the serum of several HD mouse models (IL-6, IL-10, IL-1β, IL-12p70) (Björkqvist et al., 2008), in primary cultures of HD microglia isolated from R6/2 mice (IL-6, TNFα) (Crotti et al., 2014), and in microglia derived from HD human pluripotent stem cells (PSC) (IL-6, TFNα) (O’Regan et al., 2021). This pro-inflammatory phenotype of HD microglia is linked to a cell-autonomous mechanism as specific expression of mHTT in microglia induces pro-inflammatory transcriptional activation (Crotti et al., 2014). A strategy to reduce pro-inflammatory cytokines in HD mice prevents motor impairment, restores neuronal DARPP32 levels and expands life span (Siew et al., 2019).

Overall morphology, transcriptional profile, function alteration, and pro-inflammatory phenotype of microglia might underlie neuronal degeneration in HD. Since microglia has a crucial role in synaptic pruning, maturation, maintenance, their dysfunctions could be linked to HD synaptic alteration, including electrophysiological modifications in MSN and a decreased dendritic spine density. Neuronal damages in the striatum of R6/2 mice are associated with an alteration of microglia-synapse interaction. HD microglia display a more mature morphological phenotype, increase phagocytosis across the age and make fewer contact with synaptic structure, as compared with control mice. This is related with a disruption of synaptic contact localization and synaptic density in R6/2 mice (Savage et al., 2020). Altogether, these data suggest that, like astrocytes, microglia and their interaction with other cell types in the CNS need to be considered as a contributor of HD. Indeed, the expression of mHTT in microglia has a toxic effect when co-cultured with WT neurons. Moreover, the lipopolysaccharide- induced-microglia activation leads to a higher neuronal death in HD mice as compared with control, which might be due to an over-inflammatory response in mHTT-expressing microglia (Crotti et al., 2014). Reactive astrocytes can also produce pro-inflammatory and/or anti-inflammatory molecules, and activated microglia can induce the activation of pro-inflammatory astrocytes, favoring cell death (Liddelow et al., 2017). Microglia release exosome containing RNA identified to be part of hub genes and protein networks known to have a role in neurodegenerative diseases, including HD, Parkinson’s disease (PD), AD, through immune inflammation and oxidative stress pathways (Xie et al., 2022). In parallel to the deleterious effect of mHTT in microglia for brain homeostasis, several studies focused on the consequences of the depletion of either mHTT in microglia, or HD microglia. The depletion of mHTT selectively in microglia does not rescue behavioral or neuropathological phenotype in BACHD mouse model, whereas mHTT depletion in all other cell type except microglia rescue behavioral phenotype and striatal volume (Petkau et al., 2019). Conversely, the depletion of microglia in R6/2 mice, prevents some motor and cognitive deficits, mHTT aggregates accumulation, astrogliosis, and striatal volume loss (Crapser et al., 2020).

Microglia can affect neuronal functions and survival in HD, but a bidirectional interaction and the impact of HD neurons on microglia also need to be explored. Indeed dynamic of microglia processes can be regulated by neuronal activity (Liu et al., 2019), and conditioned medium from human striatal neurons expressing mHTT is toxic for HD human pluripotent stem cell-derived microglia (O’Regan et al., 2021). The neuro-inflammation observed in HD most probably arise from a combination of cell-autonomous and non-cell autonomous activation of microglia, with a communication between damaged neurons, reactive astrocytes and microglia. The cross talk between these cellular populations is a key point to understand CNS alterations in HD.

### Cholesterol Metabolism and Microglia

So far, there are no studies demonstrating a direct link between cholesterol metabolism dysregulation and microglia alterations in HD. However, some directions might be of interest to explore such as a link between microglia and myelin deficits. Indeed, myelin contain 70% of total CNS cholesterol, and HD is associated with a decrease of myelinisation process and an age-dependent demyelination (Huang B. et al., 2015). Following myelin impairment, microglia induces the phagocytosis of myelin debris (Reichert and Rotshenker, 2003), associated with a production of pro-inflammatory cytokines. Therefore, microglia have an important role in cholesterol clearance, and is essential for maintaining cholesterol homeostasis in the brain, thus avoiding its neurotoxic accumulation. Cholesterol debris clearance by microglia could be linked to the altered activity of the nuclear receptor LXR. Indeed, HD is associated to a downregulation of LXR target genes, and a decreased level of LXR ligand (desmosterol, 24S-OHC), suggesting a reduced LXR activity. In demyelinated regions, desmosterol is synthetized in microglia during myelin debris phagocytosis, inducing LXR activation, followed by inflammation resolution, stimulation of oligodendrocyte differentiation and increased cholesterol efflux from microglia for supporting re-myelinization process (Berghoff et al., 2021). Therefore cholesterol homeostasis is important for microglia phagocytosis functions, and *in vitro* treatment of microglia with simvastatin, a cholesterol lowering drug, alters microglia phagocytosis, BDNF and inflammatory factors (IL1-β, TNFα) release (Churchward and Todd, 2014), described to be downregulated in HD (Björkqvist et al., 2008). Cholesterol is also important for microglia survival (Bohlen et al., 2017). Therefore, exploring microglial phagocytosis of myelin debris in HD could provide new insight into understanding cholesterol metabolism in HD and its involvement brain homeostasis.

## Clinical Strategies to Manipulate Cholesterol Metabolism in Central Nervous System

**FIGURE 3:**
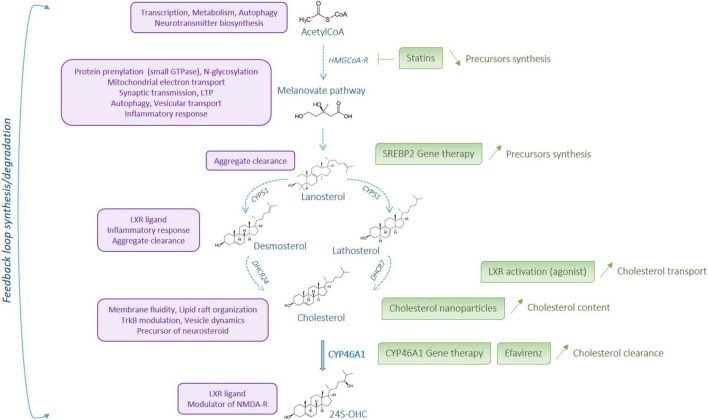
Cholesterol metabolism: a potential therapeutic target. Cholesterol metabolism steps are represented in blue. The role and action of the different metabolites are described on the left, in purple. On the right, in green, are listed some options for therapeutical intervention to restore cholesterol metabolism in Huntington’s disease. Created with BioRender.com.

Brain cholesterol content must be tightly regulated to ensure normal brain function and its dysregulation is linked to neurodegenerative diseases for not only HD but also AD and PD (Dai et al., 2021). Based on these reports, cholesterol metabolism is a potential therapeutic target for neurodegenerative diseases (Figure 3). Statins are a class of molecules used to lower cholesterol by inhibiting HMGCoA-R, the rate-limiting enzyme in cholesterol pathway synthesis. These molecules, which are capable of crossing the blood-brain barrier, confer suitable drugs to regulate brain cholesterol. Over the past decade, preclinical and clinical studies using statins in AD and PD have been reported with controversial findings. In AD, several studies showed that statins can reduce the risk of the disease (Wolozin et al., 2000; Rea et al., 2005; Sparks et al., 2005; Feldman et al., 2010; Lin et al., 2015) whereas no therapeutic effect was described in other studies (Rea et al., 2005; Feldman et al., 2010). The clinical studies in PD have shown that the use of statins can reduce the risk of the disease (Bai et al., 2016; Sheng et al., 2016), whereas other studies describe no effect of these molecules (Huang X. et al., 2015; Liu et al., 2017; Rozani et al., 2017; Lin et al., 2021). If statins are primary used to lower cholesterol content, these molecules also show anti-inflammatory, anti-oxidative, and anti-excitotoxic properties that need to be considered in therapeutic options for neurodegenerative diseases, especially in HD. Lowering sterol levels by statin treatment is not intuitive in HD clinical strategy as the content of cholesterol precursors and enzyme activity are decreased within the brain. However, statin assay can be relevant to reduce the inflammatory response, oxidative stress and excitotoxicity that occur in HD. In HD cellular models, administration of simvastatin protected against NMDA mediated excitotoxicity by reducing the content of lipid rafts domains in the plasma membrane of mHTT cells (del Toro et al., 2010). In quiescent HD fibroblasts an impairment of the ATM-dependent signaling and repair pathways of the DNA double-strand breaks by the non-homologous end-joining (NHEJ) repair process has been described after ionizing radiation (Ferlazzo et al., 2014). In this study, a combination treatment with statin and bisphosphonate, which inhibits membrane farnesylation of the nucleus-, increased the nucleo-shuttling of ATM kinase and improved DNA double-strand repair by NHEJ. In the experimental HD rat models induced by quinolinic and malonic acids or 3-NP, simvastatin showed beneficial effects on HD phenotype (Patassini et al., 2008; Ahmed et al., 2016). In the quinolinic acid HD rat model, simvastatin induced immunoreactivity for Bcl-2, an anti-apoptotic factor, on one hand, and down-regulated immunoreactivity for Bax, a pro-apoptotic factor (Patassini et al., 2008). Atorvastatin and simvastatin treatment alleviated malonic acid induced HD like symptoms and related cognitive dysfunctions (Kumar et al., 2013). In particular, the motor behavior was improved, with reduced oxidative stress, restoration of mitochondrial dysfunction, increased mitochondrial complex I, II, III, and IV activities, and reduced neuro-inflammation accompanied by a decrease of TNF-α and IL-6 levels. In the 3-NP HD model, simastatin can decrease neurotoxicity, mitochondrial dysfunction through SDH activity regulation and inflammatory response by modulating the nitric oxide synthases eNOS and iNOS activity (Ahmed et al., 2016). Electrophysiological studies addressed the effects of simvastatin on striatal activity of MSNs from symptomatic R6/2 mice and showed an increased frequency of spontaneous inhibitory postsynaptic currents compared with controls. Simvastatin treatment decreased sIPSCs of R6/2 slices through a mechanism that needs to be determined (Chen et al., 2016).

So far, no clinical trials have been conducted to evaluate the effect of statin in HD. One study used the Enroll-HD database to investigate if statin use among patients with premotor HD can be associated with beneficial effects (Schultz et al., 2019). In patients with premotor HD, statin use was associated with a delayed motor diagnosis of HD suggesting that statin may provide a neuroprotective benefit in the early course of HD. It seems reasonable to encourage clinical trials of statins in HD because of their effects on modulating the inflammatory response and oxidative stress rather than their ability to regulate cholesterol metabolism.

As mentioned above, reduced synthesis of brain cholesterol precursors is well described in the literature whereas the cholesterol content is the subject of controversy: studies reported an increased accumulation of free cholesterol whereas a decrease of cholesterol content is also described (Table 1). The temporal alterations of cholesterol metabolism needs to be considered as HD progresses. Dietary supplementation to regulate cholesterol metabolism has been performed using anthocyanin and Manganese (Kreilaus et al., 2016a; Pfalzer et al., 2019). Anthocyanin is highly concentrated in berry extracts which improved cognitive function in rodents during aging (Galli et al., 2006; Duffy et al., 2008). However, anthocyanin supplementation did not influence disturbances to cholesterol synthesis whereas it improves motor performance in R6/2 mice (Kreilaus et al., 2016a). Manganese is one variable which may impact cholesterol biosyntheis but acute systemic exposure of pre-manifest and manifest YAC128 to manganese did not regulate cholesterol biosynthesis (Pfalzer et al., 2019). The team of Prof E. Cattaneo provided consistent studies to demonstrate the beneficial effect of striatal cholesterol infusion in HD mouse models. The first proof of concept was achieved using brain-permeable polymeric nanoparticles loaded with cholesterol, which can cross the blood-brain barrier to reach glial and neuronal cells in different brain regions. After repeated systemic administration of the cholesterol-loaded nanoparticles in R6/2 mice, synaptic and cognitive defects were rescued whereas global activity was partially improved and no effect on neuropathology was noticed (Valenza et al., 2015a). Furthermore, beneficial effects of cholesterol supply were confirmed using novel hybrid polymeric nanoparticles to favor BBB transit (Birolini et al., 2021a). A deeper characterization of cholesterol supply was later provided using osmotic mini pumps that can directly infuse cholesterol within the striatum (Birolini et al., 2020). In R6/2 mice, cholesterol infusion prevented cognitive defects, alleviated motor phenotype and improved synaptic transmission. Interestingly, cholesterol precursors such as lanosterol, lathosterol, desmosterol and the product of cholesterol catabolism 24S-OHC were enhanced following striatal cholesterol infusion. Another way of brain cholesterol delivery was achieved using an intranasal dose of liposomes loaded with deuterium-labeled cholesterol in WT mice (Passoni et al., 2020). If the benefits of cholesterol loading are well demonstrated, further studies are necessary to determine the long-term effect of cholesterol infusion using a chronic HD mouse model. The different ways of cholesterol administration need also to be compared in terms of safety, invasive procedure, and cholesterol dose and long-term beneficial effects before clinical trials.

Cholesterol metabolism in the brain is mainly regulated *via* two key transcription factors: the LXR nuclear receptors and the sterol SREBP2. As dysfunctions of LXR and SREBP2 in HD have been reported, manipulation of these transcription factors could be considered in clinical research for HD. The LXR family includes two isoforms: LXRα, which is mainly expressed in the liver and other tissues critical for peripheral lipid metabolism whereas LXRβ is prominently expressed in the brain (Whitney et al., 2002). LXR regulate the transcription of APOE and its lipidation transporters ABCA1 and ABCG1 (Figure 2) as well as the expression of the cholesterol synthesis enzymes Fdft1 (squalene synthase) and Cyp51 (lanosterol C14 demethylase) (Wang et al., 2008). Mouse genetic models generated to invalidate both isoforms showed impairment of lipid homeostasis in the brain and neurodegeneration, illustrated by nuclear and cytoplasm condensation as well as axonal demyelination (Wang et al., 2002). Specific ablation of LXRβ impaired motor coordination associated with lipid accumulation and loss of motor neurons in the spinal cord (Andersson et al., 2005). Cholesterol transport by astrocytes is less efficient in HD, with decreased expression of APOE and ABCA1. Astrocytes expressing mHTT produce and release less ApoE, affecting cholesterol transport to neurons (Valenza et al., 2010, 2015b). Reduced transport might be associated with a decreased activity of LXR. Indeed, in normal conditions, HTT acts as a co-activator for LXR transcription factors, therefore HTT mutation can potentially lead to a loss of function and thus a decrease of LXR activity (Futter et al., 2009). Manipulating LXR activity is possible using the synthetic agonists T0901317 and GW3965 and beneficial effects of these ligands have been observed on preclinical models of AD (Suon et al., 2010; Vanmierlo et al., 2011). These compounds regulate APOE, ABCA1, ABCG1 expression, decrease cognitive defects and improve brain pathology in AD mouse models. In HD, incubation of R6/2 brain slices with T0901317 reduced the frequency and amplitude of spontaneous inhibitory postsynaptic currents compared with controls (Chen et al., 2016). The same compound can partially rescue the phenotype and the expression of LXR target genes in HTT-deficient zebrafish (Futter et al., 2009). Further studies are necessary to determine LXR agonist effects on HD preclinical model. It should be noted that in addition to their role in cholesterol homeostasis, LXR activation induces an anti-inflammatory response by decreasing the expression of many pro-inflammatory genes (iNos, IL-1β, IL-6, TNFα) (Bensinger et al., 2008). This latter point favors the need to explore LXR therapeutic targets in rodent HD model as inflammation is associated with HD pathogenesis and cholesterol metabolism is linked to neuroinflammation (González-Guevara et al., 2020). Manipulating LXR activity offers new therapeutic strategies in HD but the clinical use of these compounds is limited to the peripheral hypercholesterolemia/hypertriglyceridemia undesirable side effects. To counteract this limitation, new compounds and tools need to be designed with better brain specificity and with limited lipogenic effects attributed to LXRα in peripheral tissues (Stachel et al., 2016; Navas Guimaraes et al., 2021).

SREBP2 regulates cholesterol biosynthesis through the expression and activation of cholesterol biosynthesis genes (Figure 2). SREBP2 is expressed in glial cells, especially in astrocytes and oligodendrocytes, where it controls lipid synthesis (Camargo et al., 2009). Mutant HTT decreases the activity of SREBP2 by 50% in cells and mouse brain tissues (Valenza et al., 2005), and overexpression of its active form in astrocytes, *in vitro* and *in vivo* in R6/2 mice, showed beneficial effects in neurons (Valenza et al., 2015b; Birolini et al., 2021b). This suggests an important dialogue between glial and neuronal cells for proper cholesterol homeostasis. In this way, in an elegant study, a gene therapy approach was used to express the transcriptionally active form of human SREBP2 specifically in striatal astrocytes of R6/2 mice (Birolini et al., 2021b). The authors found a re-activation of the cholesterol pathway biosynthesis, associated with restoration of synaptic transmission, clearance of mHTT aggregates, and improvement of motor defects and cognitive decline.

### Focus on CYP46A1 as a Therapeutic Option in Huntington’s Disease

A critical role of CYP46A1 in cholesterol turnover and neuronal function, through regulation of cholesterol precursors, was well described in *Cyp46a1^–/–^* mice. In this mouse model, brain cholesterol levels were unchanged but cholesterol synthesis was reduced by 40%, potentially to compensate for the lack of degradation, showing the importance of CYP46A1 in cholesterol turnover (Lund et al., 2003). *Cyp46a1*^–/–^ mice present severe cognitive deficiencies, with impairment of spatial, associative and motor learning, associated with deficiency to establishing LTP in the hippocampus. The effects on LTP were reversed by treatment with geranylgeraniol, a precursor of cholesterol from the melanovate pathway, but not by adding cholesterol, showing the importance of cholesterol turnover, with a specific and quick action of the melanovate pathway (Kotti et al., 2006, 2008). The phosphorylation levels of many proteins was altered in the brain of these mice, including GTPase proteins (RAB8, CDC42, RAC), microtubules associated and neurofilaments proteins (MAP and NEF) along with proteins involved in synaptic vesicles formation and neurotransmission (SLCs, SHANKs). Moreover, ubiquitination is increased in proteins important for cognition, cytoskeleton function and energy production (Mast et al., 2017). By contrast, the brain of transgenic mice overexpressing human CYP46A1 (C46-HA) showed increased production of 24S-OHC and enhanced synthesis of cholesterol synthesis, with higher levels of lanosterol, consistent with increased cholesterol turnover by CYP46A1 (Shafaati et al., 2011). Interestingly, the C46-HA transgenic mice showed improvement of spatial memory with increased levels of proteins involved in neurotransmission: PSD-95, synapsin-1, synaptophysin, GluN1 and phosphorylated GluN2A subunit of NMDA-R (Maioli et al., 2013). CYP46A1 overexpression in neurons *in vitro* increases neuronal dendritic outgrowth and protrusion density, associated to an enhancement of synaptic proteins in synaptosomal fractions (Moutinho et al., 2015). These effects are dependent on geranylgeranyl transferase-I (GGTase-I) activity, which in turn increase HMGCR activity and membrane levels of sGTPase Rac1, Cdc42, Rab8, and RhoA. This increase in membrane sGTPases is also observed *in vivo*, in transgenic C46-HA mice (Moutinho et al., 2015). Reduction of cholesterol content by CYP46A1 is the trigger for increased phosphorylation of TrkB receptor, TrkB interaction with GGTase-I, which allows GGTase-I activity, and consequently dendritic outgrowth. These results were replicated *in vivo*, with an increase of p-TrkB and synaptic proteins in synaptosomal fractions prepared from CYP46A1 transgenic mouse cortex (Moutinho et al., 2016). Overall, CYP46A1 has an essential role in synaptic functions by stimulating cholesterol turnover, particularly through the melanovate pathway.

Overall, these findings indicate that CYP46A1 is an interesting therapeutic target to consider for the treatment of neurological disorders. Levels of 24S-OHC, which are directly correlated to CYP46A1 activity, are changed in several neurodegenerative diseases such as PD (Björkhem et al., 2013), AD (Bretillon et al., 2000; Lütjohann et al., 2000; Schönknecht et al., 2002), multiple sclerosis (Teunissen et al., 2003), dementia (Kölsch et al., 2004), and HD (Leoni et al., 2008). Because of these links between CYP46A1 and neurodegenerative diseases, therapeutic strategies focusing on CYP46A1 activity have been explored in preclinical studies (Petrov et al., 2019a,b). We refer to the review of Cartier and Pikuleva for further details in AD and PD (Pikuleva and Cartier, 2021). In HD, CYP46A1 expression is decreased in the post-mortem putamen of patients and the striatum of R6/2 and zQ175 mouse models (Kreilaus et al., 2015, 2016b; Boussicault et al., 2016; Kacher et al., 2019). In the WT context, knocking down CYP46A1 expression in the striatum, *via* an adeno-associated virus-mediated delivery of selective shCYP46A1, reproduced the HD phenotype, with spontaneous striatal neuron degeneration and motor deficits, as assessed by rotarod (Boussicault et al., 2016). A recent strategy of gene therapy delivery of CYP46A1 in the striatum of R6/2 and zQ175 mice allowed an improvement of neuronal atrophy, a decrease of mHTT aggregates and improved motor behavior (Boussicault et al., 2016; Kacher et al., 2019). In these mice, cholesterol metabolism was enhanced, with not only an increase of cholesterol degradation (decrease content of cholesterol, and increased production of 24S-OHC), but also an increase of cholesterol precursors content (lanosterol, desmosterol), and expression levels of cholesterogenic enzymes (HMGCR, FDFT1, CYP51, DHCR24, DHCR7) and APOE. Importantly, in HD zQ175 mice, CYP46A1 broadly affects the transcriptomic signature related to major pathways altered in HD, including synaptic transmission, vesicular transport and unfolded protein metabolism. This new transcriptomic signature showed a global compensation of altered functions in HD through stimulation of cholesterol metabolism, causing a global restoration of striatal neurons dysfunctions, including neurotransmission, spine density and axonal transport. Other studies support the role of CYP46A1 overexpression in alleviating HD phenotypes. Indeed, in a neuroblastoma culture model of HD, CYP46A1 over-expression reduces the quantity and size of mHTT aggregates, as well as the levels of mHTT protein, potentially through the activation of autophagy (Nóbrega et al., 2020). Interestingly, CYP46A1 overexpression protects against NMDA-mediated excitotoxicity in a cellular model of HD (Boussicault et al., 2018). Therefore, CYP46A1 appears like an interesting therapeutic target as a neuroprotective strategy for the treatment of HD. Consideration of CYP46A1-based gene therapy in HD are also of interest, especially since vector delivery strategies in patient brain have considerably improved (Piguet et al., 2021).

## Discussion

Numerous studies confirm the promising strategy of targeting cholesterol homeostasis in HD. Now it is key to define the most appropriate approach, several being currently under investigation, from gene therapy to pharmacological molecules. Optimization of these approaches is a major challenge to achieve a successful treatment for HD patients and some major pressing questions need to be addressed in this regard:

(1) How cholesterol metabolism is regulated in pathological conditions and what is the consequence on glial cell and neuron metabolism? Given that cholesterol metabolism is impaired in HD, it is of interest to determine whether other metabolic regulations occur to compensate and in which cells. In this way, the role of astrocyte-neuron substrate shuttle in fueling neurons is well described for glucose oxidation (Pellerin and Magistretti, 2012). A clearer view on the interaction between the different cell types and metabolic pathways will facilitate the elaboration of more precise treatment.
(2) What are the consequences of cholesterol metabolism restoration, especially in regards to re-instating cell homeostasis in an HD context? Since mHTT is expressed throughout development and life, compensatory processes will likely occur to mitigate deleterious effects of this mutation. In particular, protein clearance is a major cellular compensatory response to face mHTT-mediated toxicity. For instance, autophagy may be an essential factor for neuronal homeostasis maintenance and its impairment has been reported for the development of HD (Martin et al., 2015). Restoration of cholesterol metabolism through CYP46A1 activity promotes protein clearance likely by induction of autophagy (Kacher et al., 2019; Nóbrega et al., 2019, 2020). Other cell compensatory mechanisms, described in HD, should be considered such as anti-oxidative stress response and DNA damage response to determine whether cholesterol metabolism can also affect these processes.
(3) How cholesterol precursors and cholesterol catabolism products can influence HD pathogenesis? The modulation of cholesterol metabolism can regulate cellular processes at different levels. Indeed, cholesterol precursors from the melanovate pathway are involved in protein prenylation, production of dolichols, heme A and Ubiquinone (Moutinho et al., 2017). Protein prenylation is essential for synaptic transmission and intracellular signaling. Dolichols function as a membrane anchor for glycoproteins. Cholesterol itself participate to membranes fluidity, organization of lipid rafts, vesicles dynamics and decreased cholesterol content by CYP46A1 is involved in TrkB activation (Martin et al., 2008). Cholesterol precursors, including desmosterol and lanosterol are directly involved in the clearance of aggregated proteins (Zhao et al., 2015; Upadhyay et al., 2018; Kacher et al., 2019). The 24S-OHC and desmosterol are ligands for LXR and therefore can modulate gene expression. Finally, 24S-OHC is an allosteric modulator of NMDA receptors mainly involved in neurotransmission. Specific roles of these bioactive molecules in HD pathogenesis need to be studied to advance our knowledge.

(4) How cholesterol metabolism within glial cells can influence HD pathogenesis? The implication of astrocytic cholesterol metabolism dysregulation in HD has been largely described but the role of microglia and oligodendrocytes need to be explored. Astrocytes secrete a range of factors including ATP, BDNF, growth factors which maintain neuronal viability and synaptic plasticity. The specific role of astrocytic cholesterol metabolism dysregulation on neuronal viability and synaptic plasticity would bring further insights in understanding the motor, psychiatric and cognitive manifests in HD.
(5) Can altered cholesterol metabolism be a good indicator of the severity of HD? Plasma cholesterol levels are similar between control, pre-HD and HD patients. However, 24S-OHC concentrations are lower in HD patients as compared with control (Leoni et al., 2008) and parallels striatal volume reduction (Leoni et al., 2013) which make this oxysterol a good biomarker to monitor disease progression. The question is now to evaluate if this oxysterol can be proposed in addition to mHTT quantification and neurofilament light chain as a biomarker to track HD progression. From there, 24S-OHC would be an interesting marker to monitor therapeutical strategies targeting cholesterol metabolism (Sipione et al., 2002; Valenza et al., 2007a,b; Leoni et al., 2011; Ritch et al., 2012; Samara et al., 2014; Shankaran et al., 2017; Phillips et al., 2020).

## Author Contributions

RK: substantial contributions to the conception and wrote sections. JC: substantial contributions to the conception and wrote sections and introduction. CM: substantial contributions to the conception and wrote one section. SB: substantial contributions to the conception and wrote sections and discussion. All authors contributed to the article and approved the submitted version.

## Conflict of Interest

The authors declare that the research was conducted in the absence of any commercial or financial relationships that could be construed as a potential conflict of interest.

## Publisher’s Note

All claims expressed in this article are solely those of the authors and do not necessarily represent those of their affiliated organizations, or those of the publisher, the editors and the reviewers. Any product that may be evaluated in this article, or claim that may be made by its manufacturer, is not guaranteed or endorsed by the publisher.

## Abbreviations

7-DHC: 7-dehydrocholesterol
24S-OHC: 24S-hydroxycholesterol
AD: Alzheimer’s disease, APOE apolipoprotein E
AMPA: α -amino-3-hydroxy-5-methyl-4-isoxazolepropionic acid receptors
BBB: blood brain barrier
BDNF: Brain Derived Neurotrophic Factor
CNS: central nervous system
CRAC: cholesterol-interacting sequence
CYP46A1: cholesterol 24-hydroxylase
CYP51: lanosterol C14 demethylase
DHCR7: -dehydrocholesterol reductase
DHCR24: 24-dehydrocholesterol reductase
ER: endoplasmic reticulum
Fdft1: farnesyl-diphosphate farnesyltransferase 1
GFAP: Glial Fibrillary Acidic Protein
HMG-CoA: 3-hydroxyl-3-methylglutaryl-coenzyme A
HMGCoAR: 3-hydroxy-3-methylglutaryl-coenzyme-A reductase
HD: Huntington’s disease
HTT: Huntingtin
INSIG: insulin-induced protein
LXR: Liver X Receptor
LTP: long-term potentiation
mHTT: mutant Huntingtin
MSNs: medium Spiny Neurons
NMDAR: *N*-methyl -D-aspartate receptor
NPC: Niemann-Pick disease
PAT: palmitoyl-acyl transferases
PD: Parkinson’s disease
polyQ: polyglutamine
PGC-1: peroxisome proliferator-activated receptor gamma coactivator-1
PTM: post-translational modifications
SDH: succinate dehydrogenase
SRE: sterol regulatory elements
SREBP: sterol regulatory element-binding protein.
